# Supplementary material for: The Oncolytic Adenovirus XVir-N-31, in Combination with the Blockade of the PD-1/PD-L1 Axis, Conveys Abscopal Effects in a Humanized Glioblastoma Mouse Model
Source: Int J Mol Sci. 2022 Sep 1;23(17):9965. doi: 10.3390/ijms23179965 (PMC9456411; doi:10.3390/ijms23179965)
Supplement: Supplementary file 1 [file ijms-23-09965-s001.zip › Supplementary Figure Legends.pdf]

Supplementary Figure Legends

Supplementary. Figure. S1. PD-L1 status of glioma cells as determined by FACS analysis.

Supplementary. Figure. S2. Immuno-humanized mouse GBM model. A. Treatment scheme. B. First signs of GvHD were determined by the measurement of human CD45+ cells in the blood of PBMC injected mice (n=2 mice). C. Body weight of PBMC engrafted mice as indicated in B (n= 2 mice).

Supplementary. Figure. S3. Identification of DAMP release by virus infected LN-229 cells. A. LN-229 cells were infected with 50 MOI of XVir-N-31, of XVir-N-31-anti-PD-L1 or with 20 MOI of Ad-WT. Supernatants were collected at the time point the cultures showed 50% cell lysis and were analysed for YB-1, HSP70 and HMGB1 release by ELISA. B. Calreticulin surface expression was analysed as described in the methods part (XVir-PD-L1: XVir-N-31-anti-PD-L1).

Supplementary. Figure. S4. Nivolumab monotherapy did not induce effective ICD in U87MG gliomas. Immunofluorescence staining for HSP70 (A), and HMGB1 (B), representative pictures are shown (n= 7- 8 mice per group).

Supplementary. Figure. S5. A. Detection of HSP70 in U87MG ipsilateral virus-injected and contralateral untreated tumors after intratumoral injection of either PBS (sham), 3 x 108 IFU of either Ad-WT, XVir-N-31 alone or in combination with multiple systemic applications of Nivolumab, or of XVir-N-31-anti-PD-L1 (n= 7- 8 mice per group; representative pictures are shown). B. Enlightment of HSP70 staining as indicated in A.

Supplementary. Figure. S6. XVir-N-31 does not replicate in PBMCs. Human PBMCs were infected with either Ad-WT or XVir-N-31. At the indicated time points after infection genomic DNA from infected cells was prepared and viral copy numbers were determined by quantitative PCR using primers specific for the adenoviral hexon gene as described in the methods part. The reduction of virus copy numbers in XVir N-31 infected cells over time indicates the inability of XVir-N-31 to replicate in PBMCs.

Supplementary. Figure. S7. After intratumoral injection of XVir-N-31, human CD45+ cells (green) were only detected in the tumor area (indicated by human nuclei staining (red); ipsilateral tumors were shown; representative pictures depicted from the XVir-N-31 group of animals).

Supplementary. Figure. S8. Nivolumab monotherapy. U87MG bearing mice were intraperitoneally injected with Nivolumab as indicated in the methods part and in Suppl.Fig. 1 A. The number of human CD45+ TILs in ipsi- and contralateral tumors (A) as well as the tumor volume (B) was determined (n= 8 mice for in the Nivolumab group, n= 4 mice in control groups, SEM; **** $p< 0.05$, ** $p < 0.01$, *** $p < 0.001$, **** $p < 0.0001$)

Supplementary. Figure. S9. Representative photographs of tumor infiltrating T cell subtypes in ipsi- and contralateral tumors. Pictures were taken 35 days after the intratumoral injection of ipsilateral (injected) as well as of contralateral (not injected) tumors (n= 7-8 mice/group).

Supplementary. Figure. S10. Representative photographs of NK cells and Tregs in ipsi- and contralateral tumors. Pictures were taken 35 days after the intratumoral injection of ipsilateral (injected) as well as of contralateral (not injected) tumors (n= 7-8 mice/group).

Supplementary. Figure. S11. Tumor infiltrating lymphocytes after OVT in NSG mice bearing LN-229 tumors. A-D. Quantification of the infiltration of different immune cells per mm2 in the tumor on the ipsilateral as well as in the contralateral side. A: CD45+ TILs, B: CD3+ TILs, C: CD8+ TILs, D: CD4+ TILs (XVir-PD-L1: XVir- N-31-anti-PD-L1; n= 7-8 tumors and 5 slices per tumor were analyzed; SEM; ns: not significant; * $p < 0.05$, ** $p < 0.01$, *** $p < 0.001$, **** $p < 0.0001$)
