# Supplementary figures and images for: The Oncolytic Adenovirus XVir-N-31, in Combination with the Blockade of the PD-1/PD-L1 Axis, Conveys Abscopal Effects in a Humanized Glioblastoma Mouse Model
Source: Int J Mol Sci. 2022 Sep 1;23(17):9965. doi: 10.3390/ijms23179965 (PMC9456411; doi:10.3390/ijms23179965)

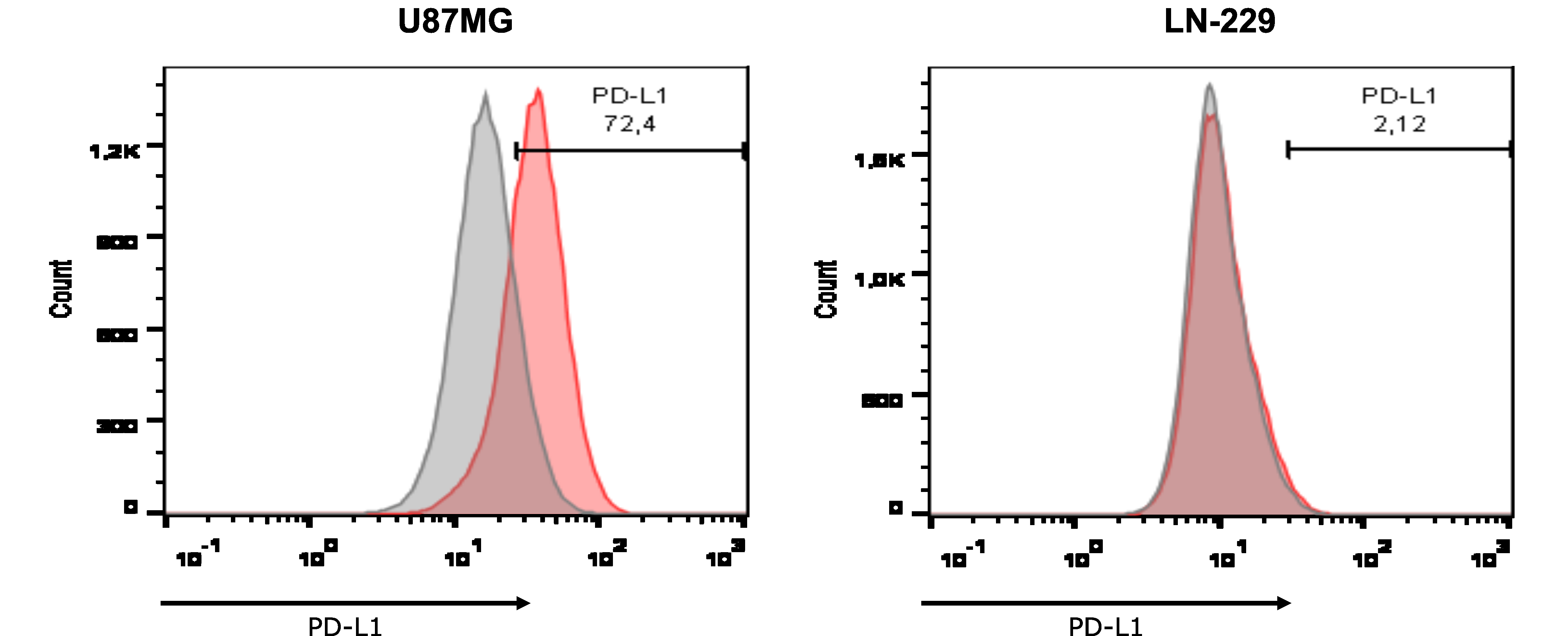

Supplement: Supplementary file 1 [file ijms-23-09965-s001.zip › Supplementary. Figure. S1.tiff]

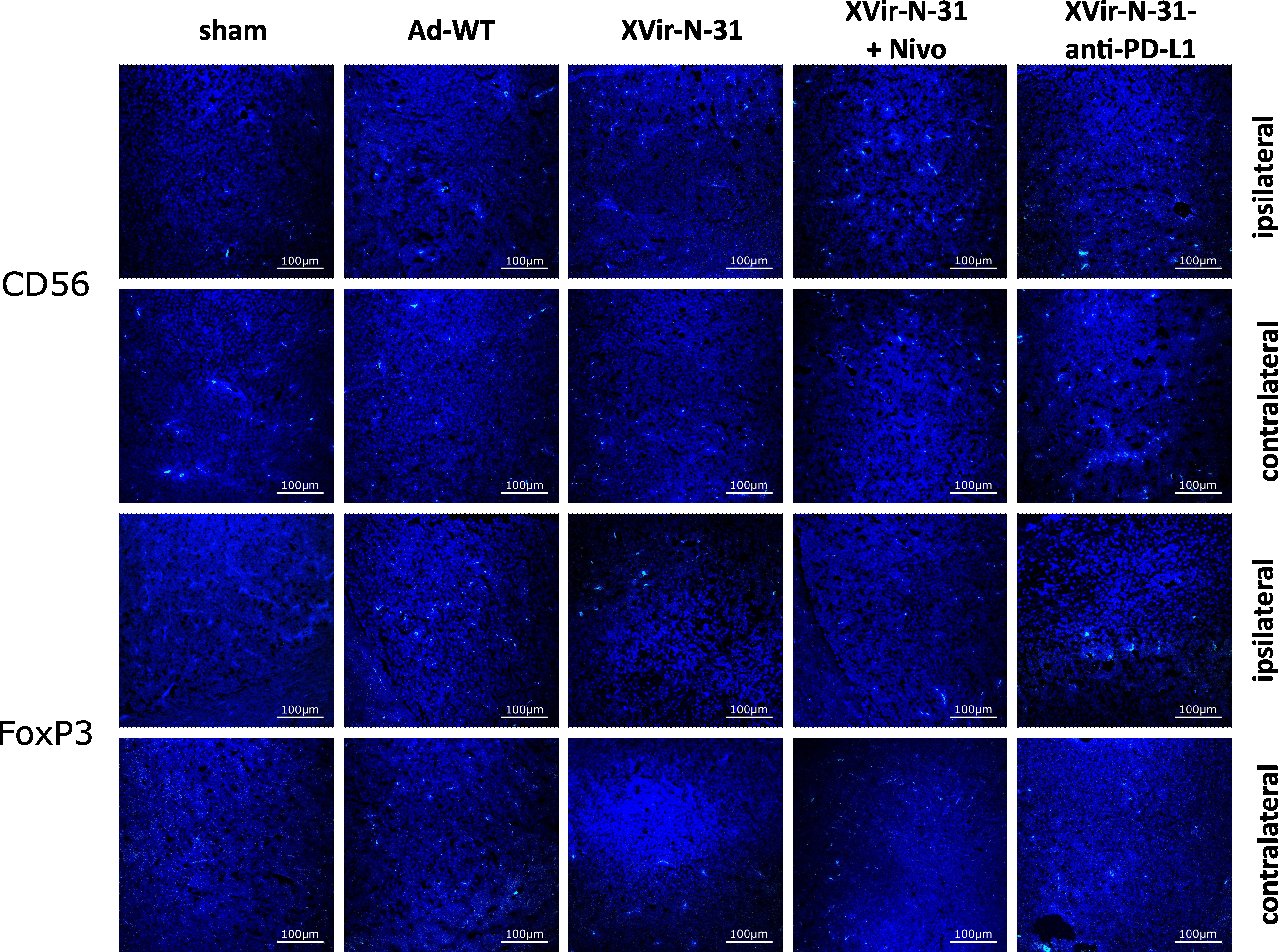

Supplement: Supplementary file 1 [file ijms-23-09965-s001.zip › Supplementary. Figure. S10.tiff]

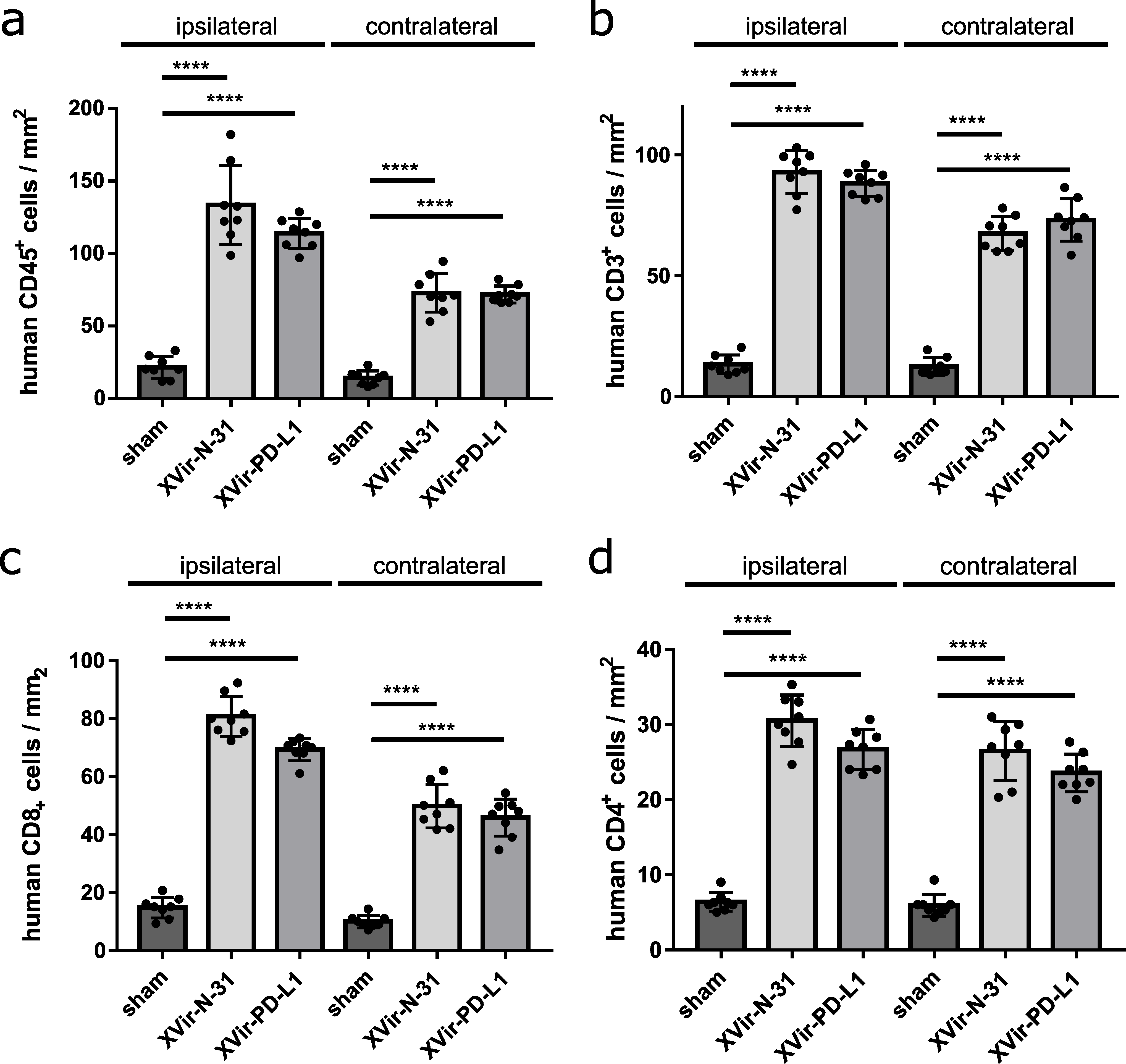

Supplement: Supplementary file 1 [file ijms-23-09965-s001.zip › Supplementary. Figure. S11.tiff]

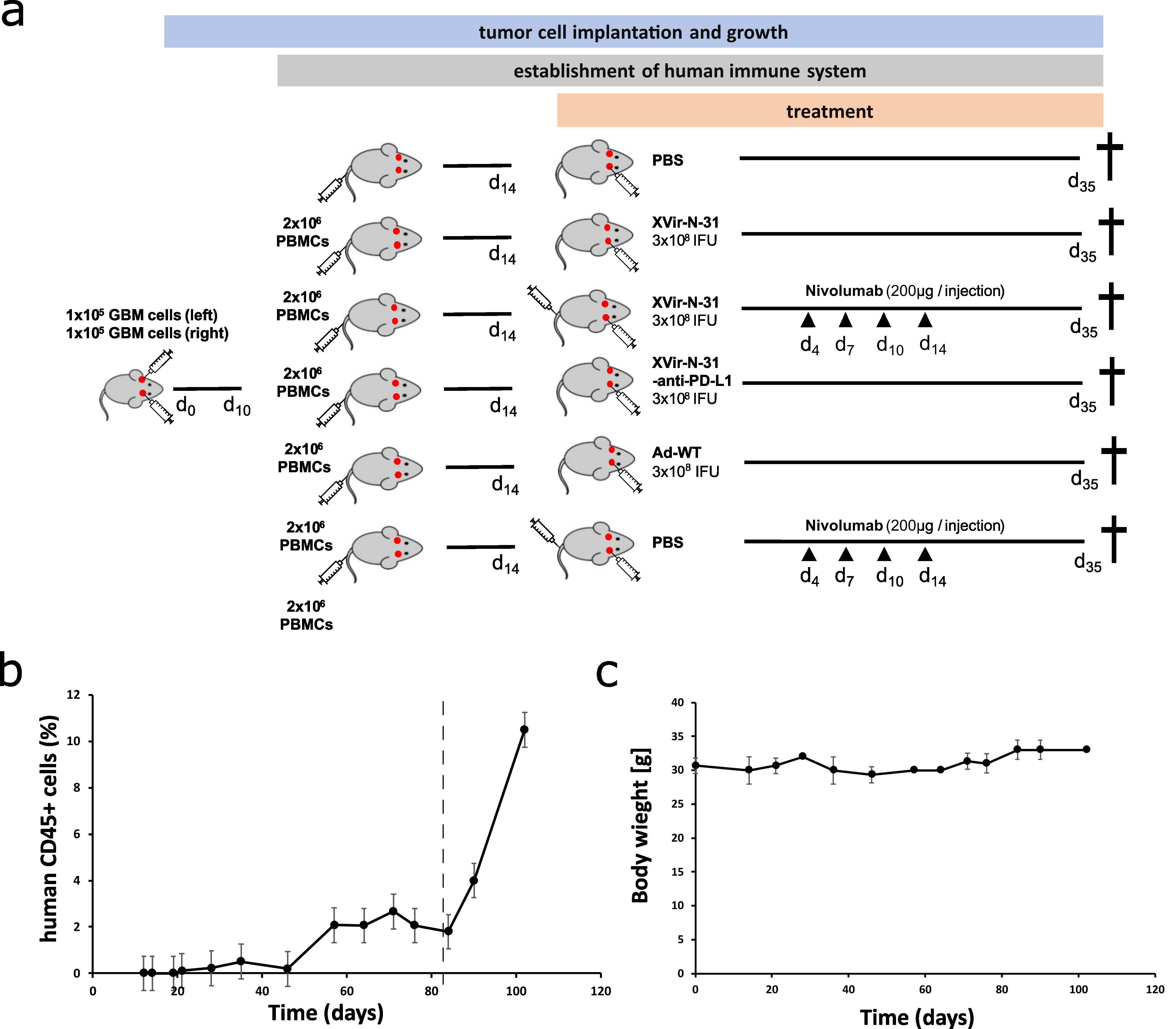

Supplement: Supplementary file 1 [file ijms-23-09965-s001.zip › Supplementary. Figure. S2.tiff]

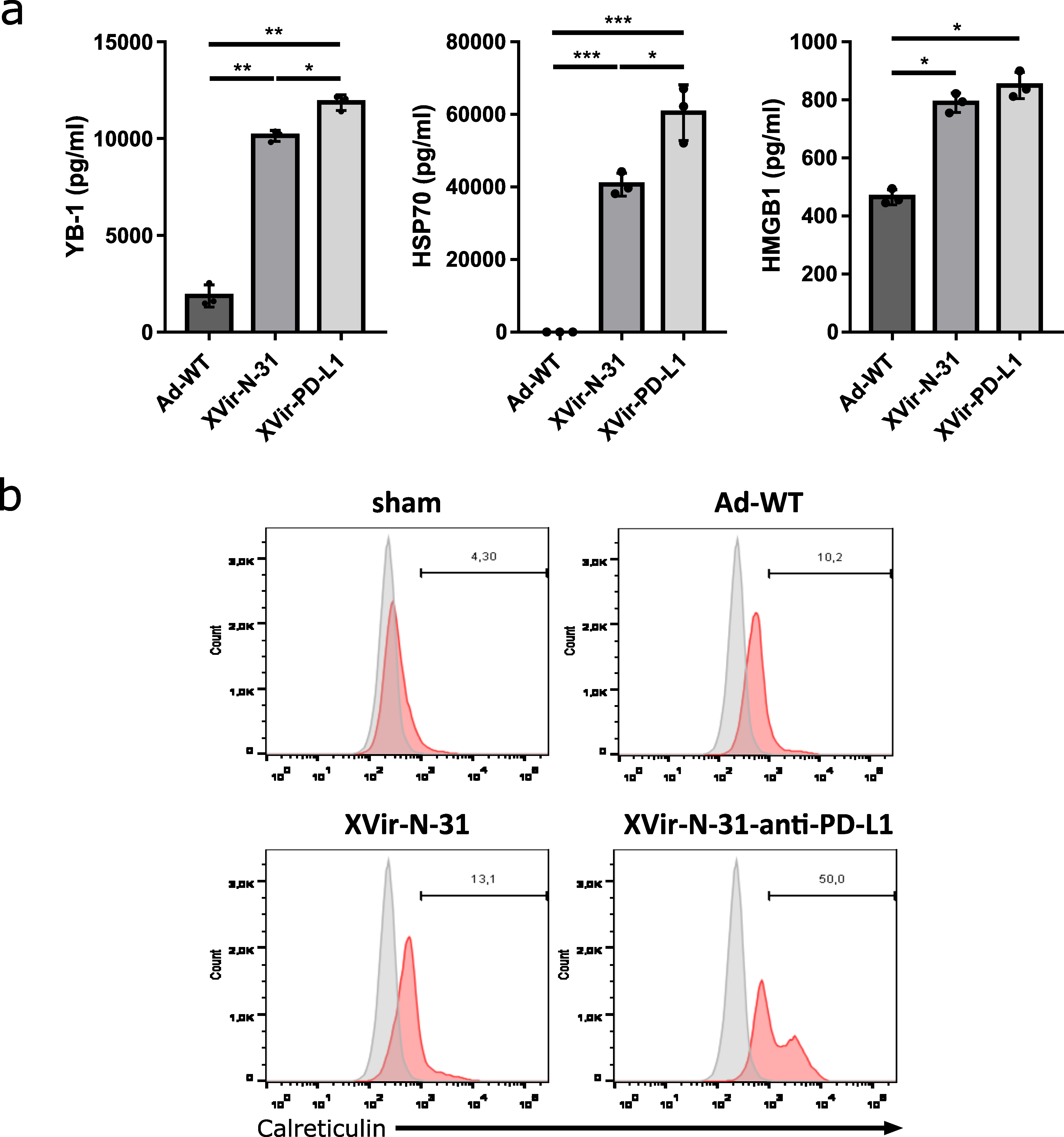

Supplement: Supplementary file 1 [file ijms-23-09965-s001.zip › Supplementary. Figure. S3.tiff]

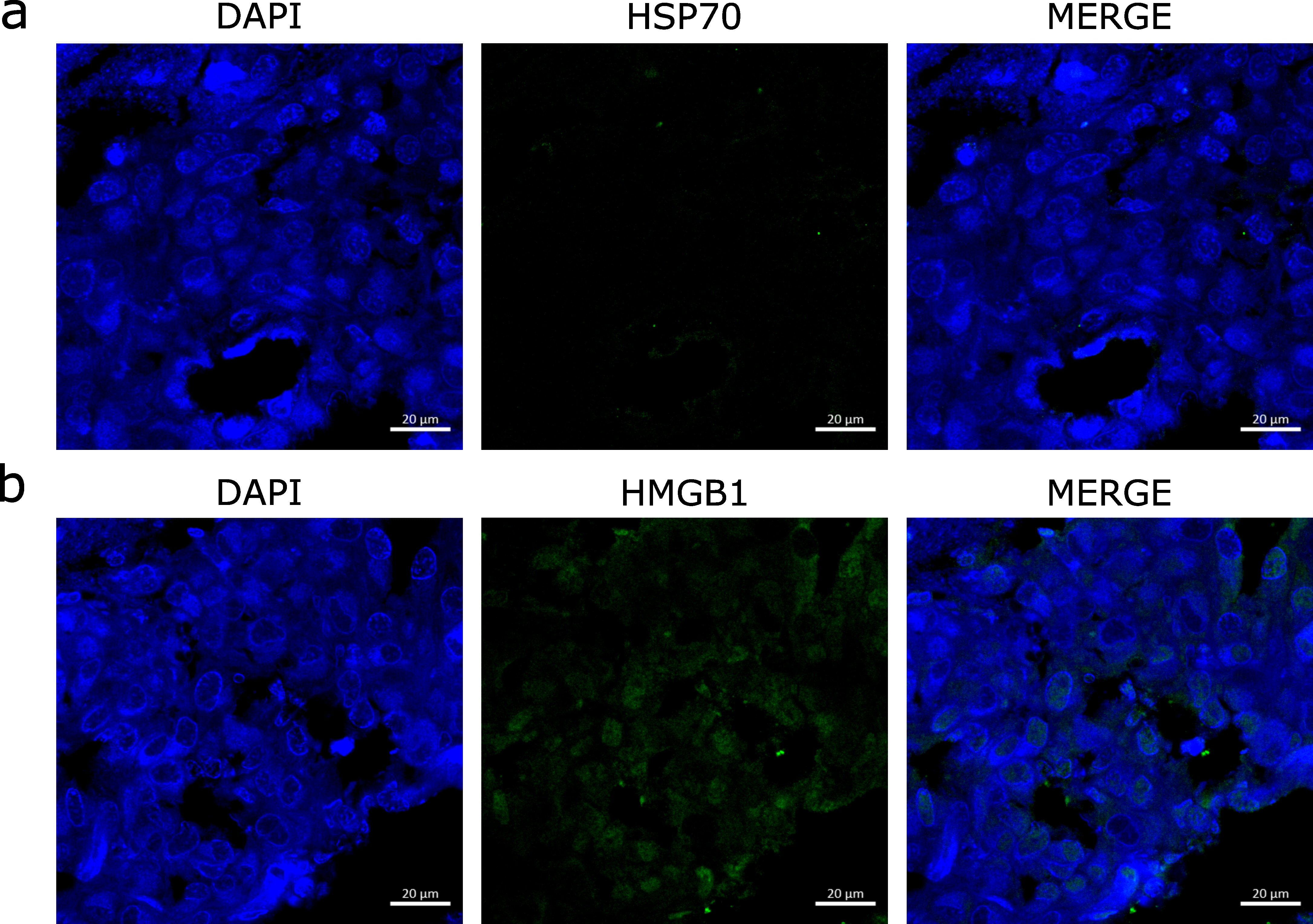

Supplement: Supplementary file 1 [file ijms-23-09965-s001.zip › Supplementary. Figure. S4.tiff]

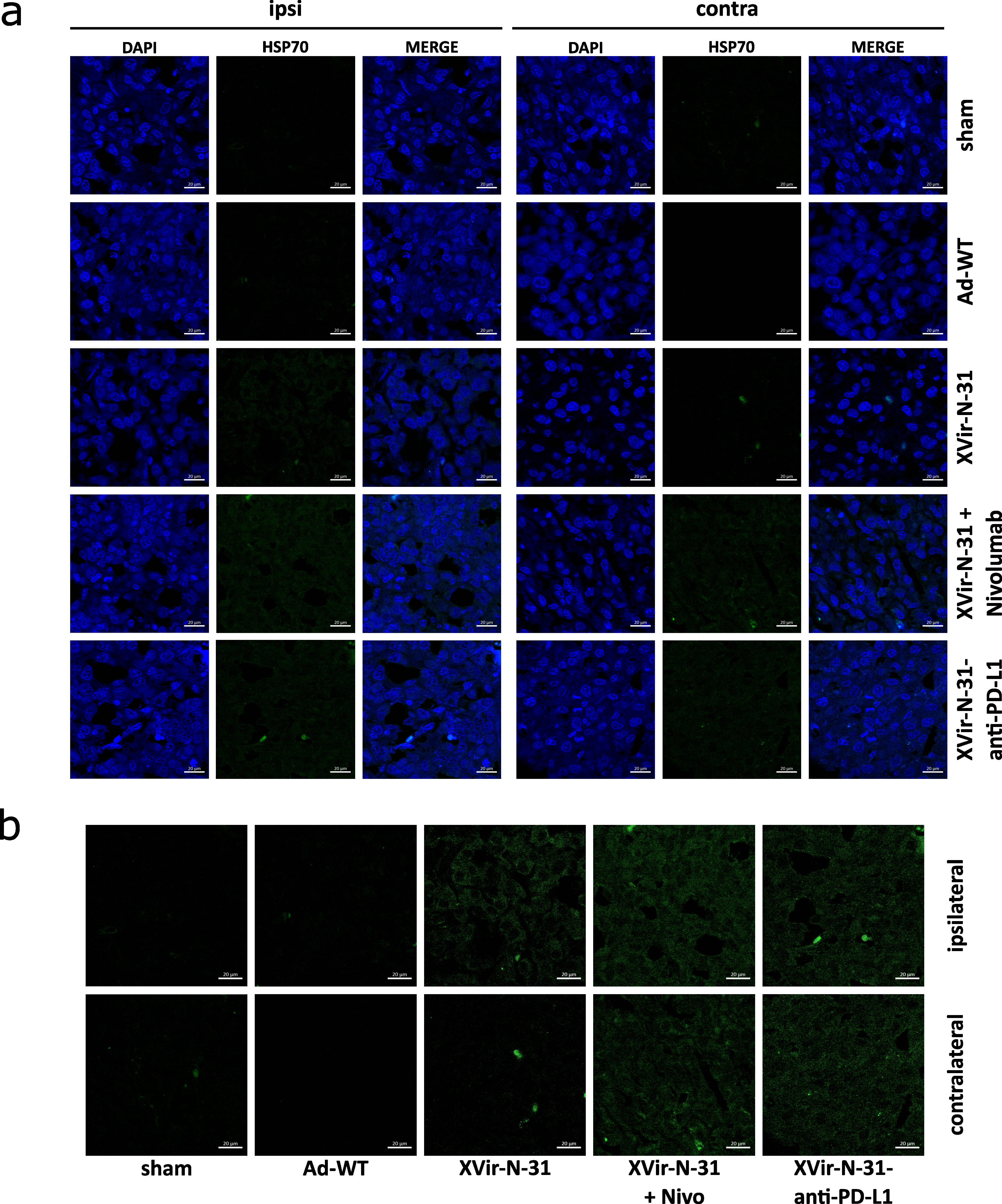

Supplement: Supplementary file 1 [file ijms-23-09965-s001.zip › Supplementary. Figure. S5.tiff]

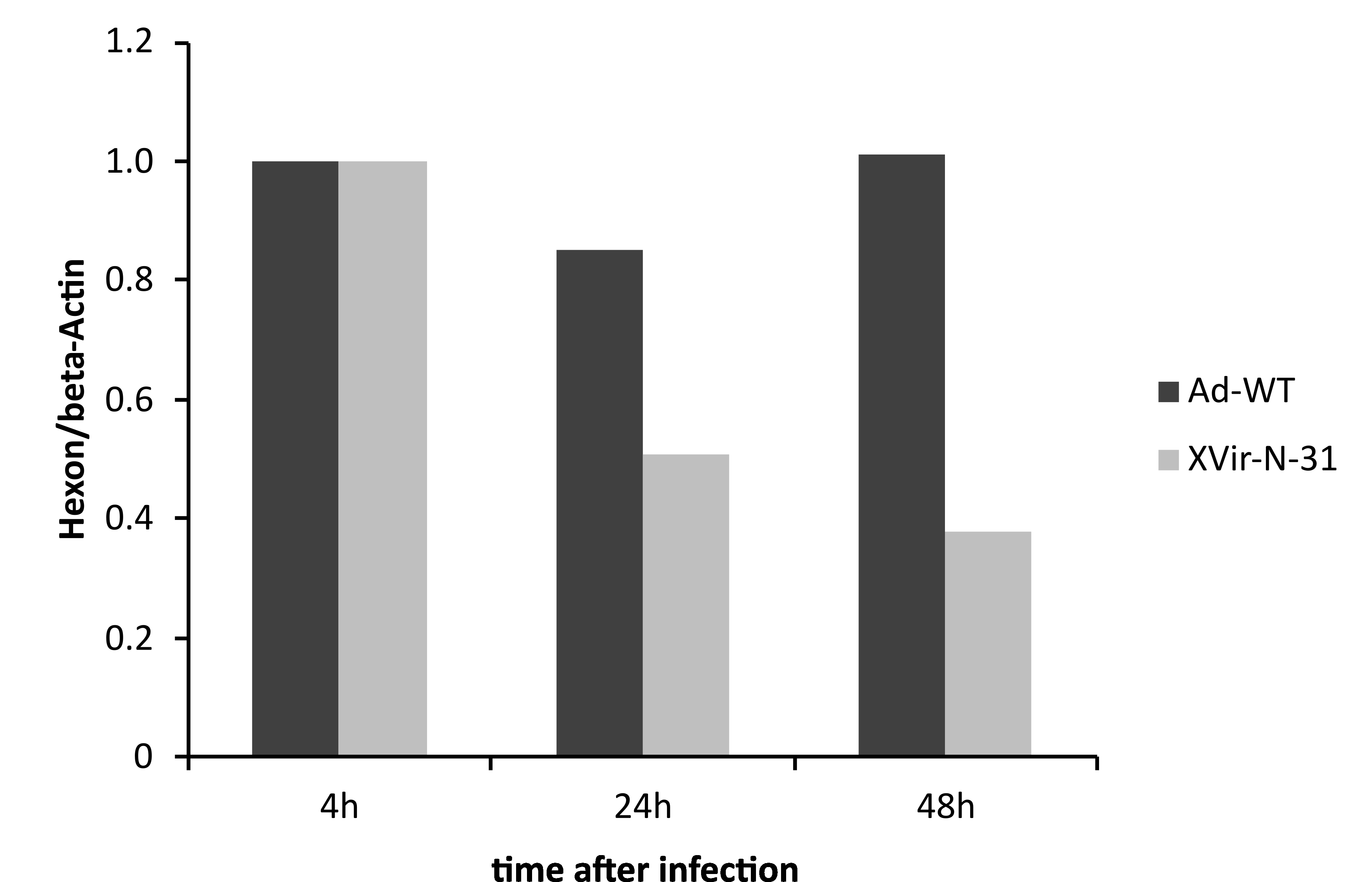

Supplement: Supplementary file 1 [file ijms-23-09965-s001.zip › Supplementary. Figure. S6.tiff]

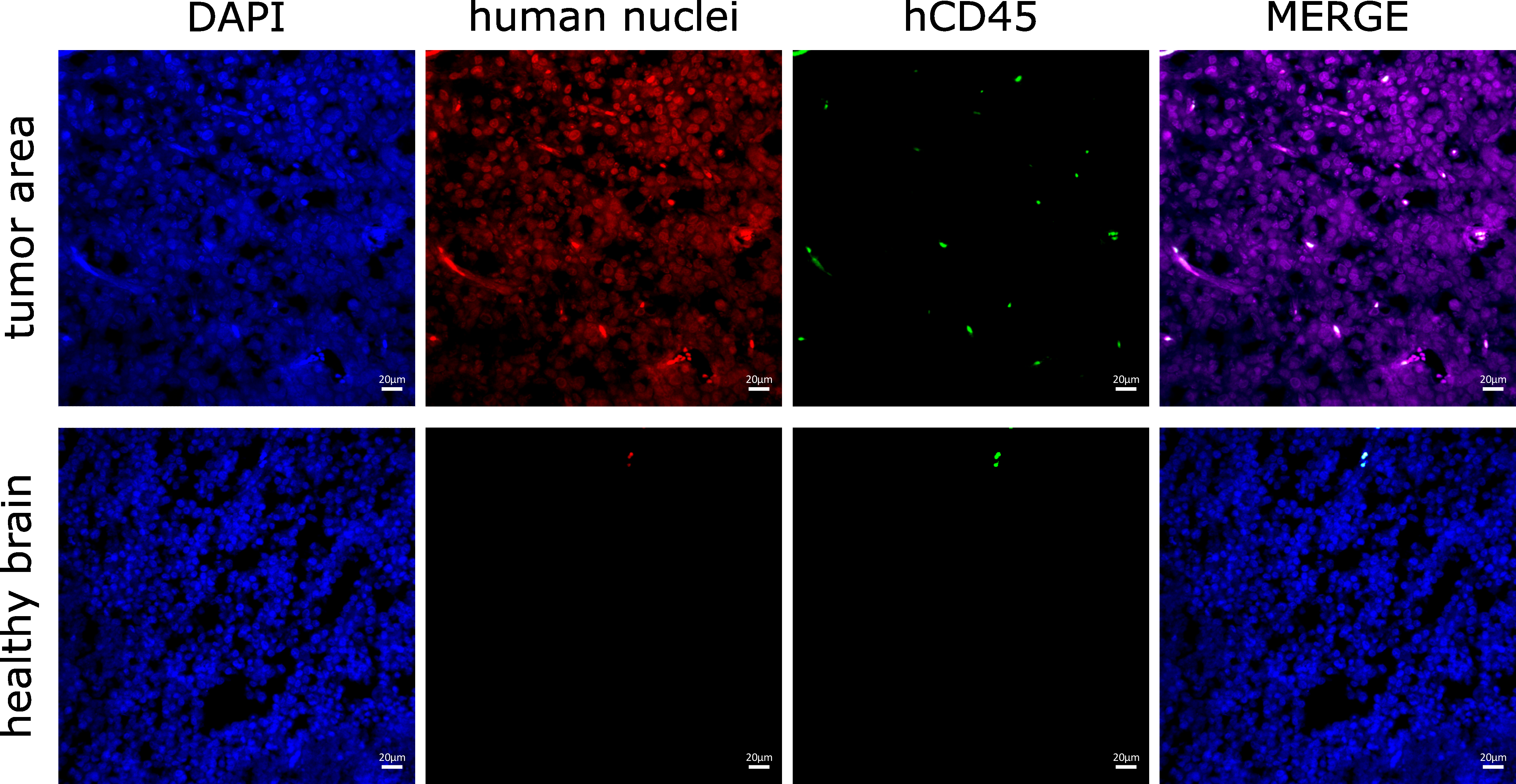

Supplement: Supplementary file 1 [file ijms-23-09965-s001.zip › Supplementary. Figure. S7.tiff]

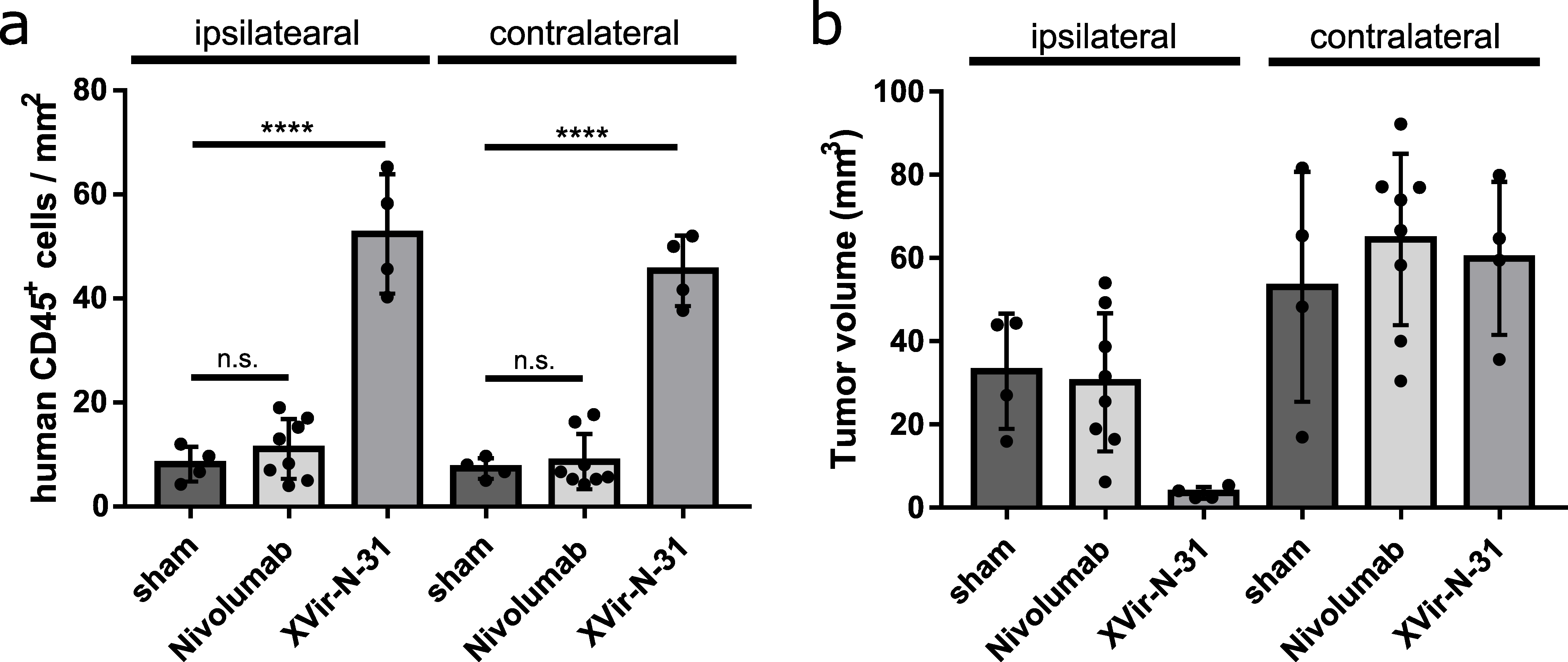

Supplement: Supplementary file 1 [file ijms-23-09965-s001.zip › Supplementary. Figure. S8.tiff]
